# Secondary syphilis in the third trimester of pregnancy

**DOI:** 10.1002/ccr3.8570

**Published:** 2024-03-03

**Authors:** Ryosuke Tajiri, Emi Kondo, Yuma Saito, Mao Sekimata, Yasuyuki Kinjo, Hiroshi Mori, Kiyoshi Yoshino

**Affiliations:** ^1^ Department of Obstetrics and Gynecology, School of Medicine University of Occupational and Environmental Health Kitakyushu Japan

**Keywords:** cesarian section/pregnancy/pregnancy complications, congenital, infectious/ syphilis

## Abstract

Syphilis infections discovered late in pregnancy, as in this case, may not be treated long enough for delivery. The Japanese guidelines should be revised because they do not describe the mode of delivery for pregnant women infected with syphilis.

## Graphical abstract

Histopathological findings in placenta of patients with syphilis.

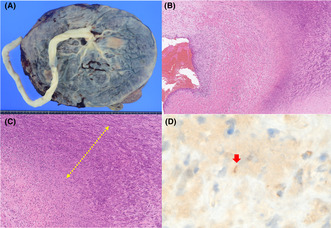

## 1 INTRODUCTION

Syphilis is an infectious disease caused by *Treponema pallidum* (TP), a member of the Spirochaetaceae family. It is one of the most common sexually transmitted diseases. According to the World Health Organization (WHO) estimates, approximately 17.7 million people all over the world between the ages of 15 and 49 years had syphilis in the year 2012, and this number reportedly increases by an estimated 5.6 million every year.^1^ The estimated prevalence and incidence of syphilis varies with region and country. The highest prevalence of syphilis has been reported in Africa, with more than 60% of new cases occur in developing countries.^1^ Gestational syphilis reportedly occurs most frequently in Africa, accounting for more than 60% of the global estimates.^2^ Syphilis is a Category 5 infectious disease in Japan according to the Infectious Diseases Law, and the number of syphilis cases has been gradually increasing since 2013.^3^ Syphilis, especially among young people, has become a concern in the recent years. Syphilis in pregnant women and congenital syphilis have become public health issues worldwide.^4^ In Japan, the hematology test for syphilis is performed at public expense as a screening test in the first trimester of pregnancy during antenatal health examination. In some foreign countries, syphilis screening tests are also conducted in the second and third trimesters of pregnancy.^5^ However, some reports indicate that screening in third trimester is unnecessary or ineffective.^6^, ^7^ In Japan, 166 cases of pregnant women with syphilis complications were reported during the five‐year period from 2012 to 2016, and 90% of these cases were detected during early pregnancy screening.^8^ Therefore, only screening in the first trimester is still being conducted, but given the current situation where the number of infected women is increasing, screening in the second or third trimester should be considered in the future.

Herein, we report a case in which a vulvar ulcer at 37 weeks of gestation led to the diagnosis of syphilis after a negative screening test during early pregnancy.

## 2 CASE HISTORY/EXAMINATION/PRESENTATION

A 32‐year‐old primipara female was referred to our hospital for painless delivery. Prenatal history included one prior spontaneous miscarriage in a year ago. The patient had a history of smoking, which she had stopped after the discovery of pregnancy. She had a history of panic disorder and bipolar disorder, for which she was on medication. However, she self‐interrupted her medication and hospital visits after discovering her pregnancy. Pregnancy was spontaneous and was managed at a primary care facility. The initial screening test performed at 12 weeks of gestation was qualitative only; it was negative for rapid plasma reagin (RPR) and TP antibodies. No abnormalities were observed in other laboratory results. At 22 weeks of gestation, she developed vulvovaginal and vaginal itching, which was diagnosed as vulvovaginal candidiasis by vaginal culture and treated with oxenazole vaginal suppositories. She was referred to our hospital at 33 weeks of pregnancy because she wanted to have a painless delivery. Thereafter, the patient continued antenatal care at the hospital. She had persistent vulvar itching and white tinea versicolor discharge. She continued the treatment for vulvar candidiasis with an oxazol vaginal suppository, but the treatment was refractory. At the time of her visit at 37 weeks and 2 days of pregnancy, painless induration and ulcerative lesions were observed on the vulva, and a blood test was performed on the suspicion of syphilis. Early in the morning of the next day, 37 weeks and 3 days after conception, she developed fever and pain during urination and was urgently admitted to the hospital on the same day.

**FIGURE 1:**
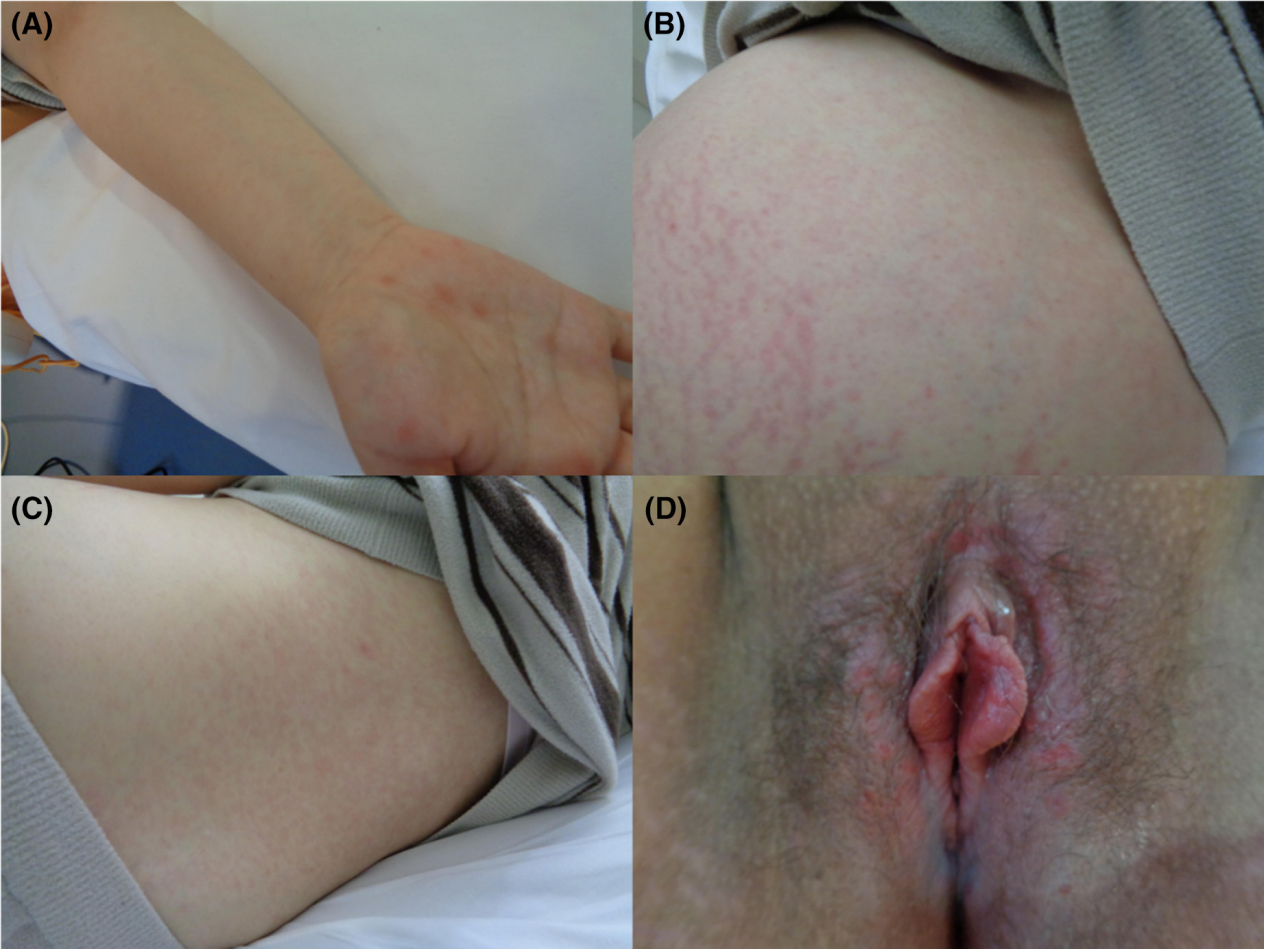
(A) Rice‐sized erythematous plaques on the palms. (B) Pale edematous erythema of the abdomen. (C) Pale edematous erythema on the back. D. Numerous rice‐sized painless ulcerative lesions on the vulva.

On admission, the patient had a fever of 37.5°C. On examination, rice‐sized erythematous plaques were observed on the palms, along with pale edematous erythematous plaques with infiltrates on the right forearm and trunk, and numerous rice‐sized painless ulcerative lesions on the vulva (Figure 1). Internal examination revealed no bleeding or water breakage, and the cervix was closed. Ulcerative lesions suspicious for syphilis were not grossly visible on the mucosa of the vaginal wall, cervix, or anus. Uterine tenderness was not observed and no symptoms of neurosyphilis were observed. Blood tests revealed a white blood cell count of 9000/μl (84.6% neutrophils); C‐reactive protein (CRP), 10.47 mg/dL; PT, 13 s, and APTT, 64 s. Cardiotocography (CTG) findings on admission were 145 bpm at baseline, moderate baseline fibrillation, transient tachycardia, no transient bradycardia, and no uterine contractions. Transabdominal ultrasonography showed that the fetus was growing at a rate equivalent to the number of weeks. None of the findings were suggestive of congenital syphilis, such as obvious fetal hepatomegaly, ascites, fetal edema, or placental thickening.

## 3 METHODS (DIFFERENTIAL DIAGNOSIS, INVESTIGATIONS AND TREATMENT)

Based on positive RPR and TP antibodies, along with clinical symptoms such as painless ulceration of the vulva and a generalized rose rash, she was diagnosed with second‐stage syphilis. Subsequent detailed interviews revealed that her husband had used a sex establishment at around 15 weeks of gestation and also had sexual intercourse with her during the same time. The patient's husband was treated with oral benzylpenicillin. After discussing the treatment plan with the Department of Infectious Diseases, the patient was treated for secondary syphilis with 4 million units of penicillin G six times a day for 10 days. The patient was in full‐term labor at the time of treatment initiation. No Jarisch‐Herxheimer reaction was observed.

## 4 CONCLUSION AND RESULTS (OUTCOME AND FOLLOW‐UP)

Although the generalized rosacea and vulvar hard chancre had resolved, the blood tests were positive for RPR and TP quantitative tests (RPR: 11.58 R.U. and TP antibody: 188.5 COI). No specific guidelines have been established for the mode of delivery in cases of syphilis diagnosed in the third trimester of pregnancy. Since the patient remained positive for RPR and TP antibodies and the risk of peripartum infection could not be assessed, we opted for a caesarean section. An emergency caesarean section was performed under spinal anesthesia at 38 weeks and 3 days of gestation due to the onset of labor pain. A girl child weighing 2908 g, with an Apgar score of 8/9 and an umbilical artery blood pH of 7.280, was delivered. The first cry was rather weak, and prompt resuscitation was initiated. Since spontaneous respiration was weak, continuous postive airway pressure (CPAP) and oxygen were administered, after which the respiratory condition improved. Based on the maternal condition, the patient was admitted to the NICU for close examination and treatment of congenital syphilis. Blood test at birth showed a negative RPR of 0.49 R.U., positive TP antibody of 18.3 COI, and FTA‐Abs‐IgM of less than 5. There was no convincing finding of fungal infection in the infant. Although there were no findings suggestive of congenital syphilis on physical examination, a dose of 50, 000 U/kg/dose of penicillin G was started after birth to prevent congenital syphilis. Vital signs of the newborn remained stable after the start of treatment. No obvious signs of infection were observed, and penicillin G administration was terminated on the 10th day of life. Early congenital syphilis test was negative, and the patient was discharged on the 19th day of life. At follow‐up, no new symptoms appeared in the child. In addition, the child was confirmed RPR‐negative at 3 months of age.

**FIGURE 2:**
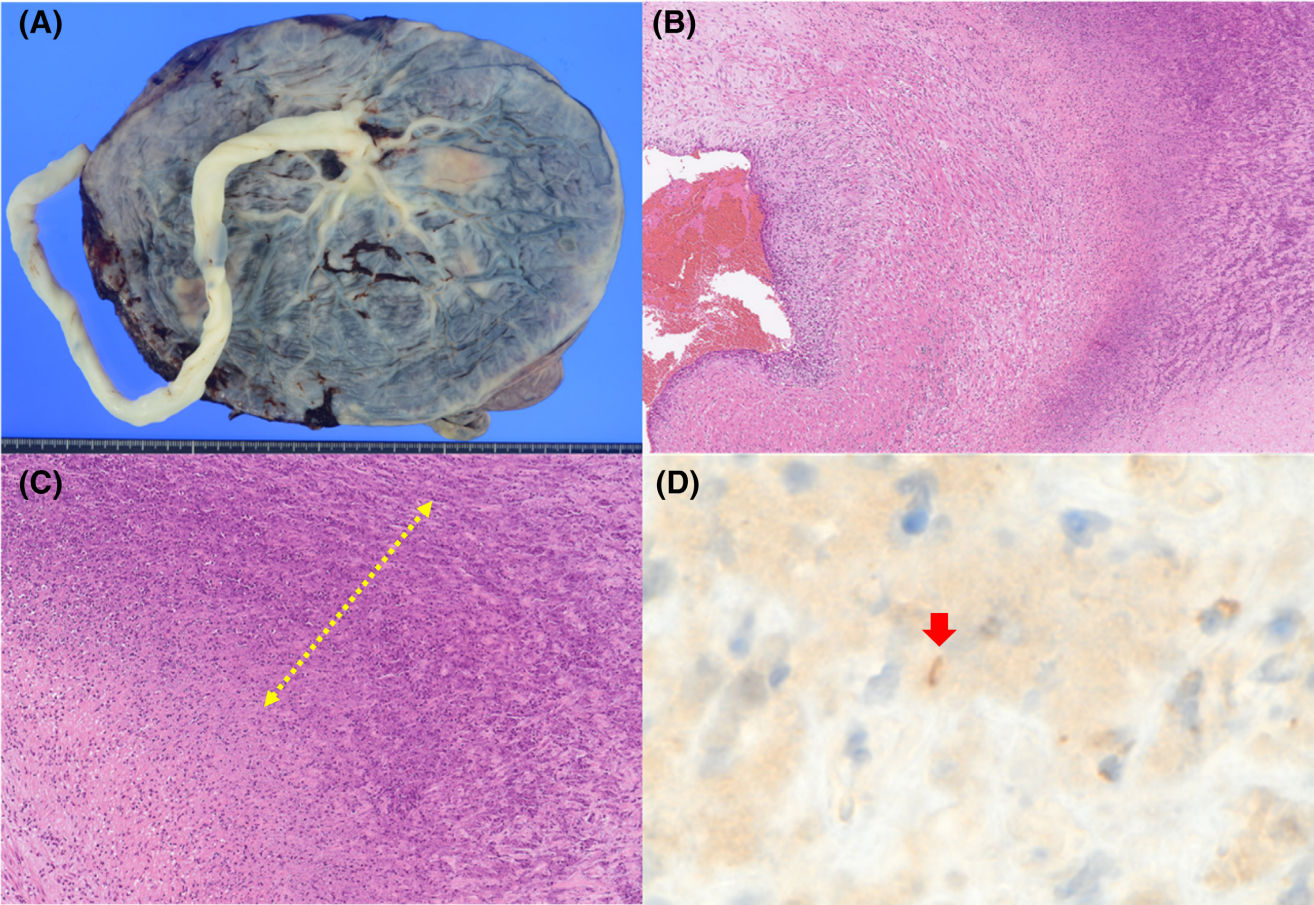
(A) Gross findings of the placenta and the umbilical cord. (B) Necrotizing funisitis (H&E). Low power (×50) showing scattered inflammatory cells in the vessel wall. (C) Necrotizing funisitis (H&E). Medium power (×200) showing scattered inflammatory cells in the vessel wall and an outer band of mild coagulation necrosis with cellular debris (dashed arrow). (D) Immunohistochemical staining for TP antibodies. High power (×400) showing slightly positive organisms (arrow).

The mother continued postoperative penicillin G infusion until postoperative day 5, after which she was discharged. The patient is currently receiving treatment on an outpatient basis. Histopathological examination of the placenta revealed necrotizing funisitis (Figure 2). In addition, immunohistochemical staining for TP antibodies showed slightly positive organisms (Figure 2).

## 5 DISCUSSION

There is insufficient literature on delivery of pregnant women with syphilis. No specific guidelines have been established regarding treatment methods or modes of delivery in syphilis patients. Consequently, treatment policies and modes of delivery vary from one medical facility to another. We reviewed the literature on management of pregnancies complicated by syphilis that occurred in the third trimester of pregnancy.

*Treponema pallidum* is a sexually transmitted spirochaete bacterium (100–200 nm by 10–20 μm) that is passed via contact with mucus membranes and during pregnancy can lead to vertical transmission and congenital syphilis in the infant.^9^, ^10^ In general, early syphilis refers to infections that can be sexually transmitted, and the WHO defines early syphilis as infections of less than 2 years duration.^11^ Patients with first episode of syphilis present with a single chancre or multiple lesions and regional lymphadenopathy, followed by secondary symptoms such as fever, headache, and maculopapular rash.^9^ Mother‐to‐child transmission primarily occurs during the primary and secondary infection periods, followed by the early incubation period.^5^, ^9^, ^12^ Infants born to infected mothers are often born premature, have a low birth weight, and exhibit clinical signs that mimic neonatal sepsis (such as poor feeding, lethargy, rash, jaundice, hepatosplenomegaly, and anemia).^13^, ^14^

Majority of syphilis cases in infants occur due to in utero transmission, although mother‐to‐infant transmission of syphilis may occur at the time of delivery. Spirochetes have reportedly been detected in placental or umbilical cord samples as early as 9–10 weeks of gestation, which substantiates transplacental transmission to the fetus.^15^

Syphilis often presents with diverse symptoms and is difficult to diagnose clinically. Painless lesions in hidden, exposed sites, such as the cervix, vagina, and rectum, are often missed. In addition, secondary syphilis eruptions and other lesions may appear hazy or could be mistaken for other diseases. In our case, the undeniable lesions of the cervix and vaginal wall were one of the factors that determined the method of delivery.

Screening for syphilis is universally recommended for pregnant women, regardless of previous exposure, because it is a highly effective preventive intervention against vertical transmission during pregnancy.^5^ Most national guidelines recommend screening for syphilis during the first antenatal visit, ideally during the first trimester. Some countries recommend that high‐risk women should be screened again in the third trimester of pregnancy and at the time of delivery to identify new infections. Repeat screening for syphilis in first and early third trimesters has been reported to be superior to single screening during the first trimester and is both cost‐effective and results in improvement in maternal and neonatal outcomes.^16^

In the present case, syphilis screening test in the first trimester of pregnancy was negative. The syphilis screening tests performed in this case were qualitative tests for RPR and TP antibodies. RPR has a sensitivity of 73%–100%, depending on stage, and a specificity of 98%. TP antibody has a sensitivity of 82–100%, depending on the stage, and a specificity of 99%.^9^ In the present case, both tests were negative in the Japanese early pregnancy screening test, which combines both tests; therefore, the patient may have been in the early stages of syphilis at the time of the test or may have contracted syphilis during pregnancy.

A detailed history after the onset of the disease suggested that she had sexually transmitted syphilis from her husband immediately before or after conceiving. Therefore, it was assumed that the initial screening test result was negative because of pre‐infection or early infection. She was in full‐term labor when the infection was discovered. Penicillin G infusion to prevent congenital syphilis was administered for 10 days, as per the package insert. The vulvar and skin lesions were confirmed to have disappeared. However, lesions on the vaginal wall and cervix were difficult to identify with the naked eye, and the possibility of transnatal vaginal infection could not be ruled out. Therefore, we elected to perform a caesarean section. There is no clear description of the mode of delivery for syphilis cases in late pregnancy in the guidelines, and this may be considered controversial. We also believe that screening for syphilis during the second trimester of pregnancy should be considered in the future.

In conclusion, we encountered a case of a pregnant woman with second‐stage syphilis complication that developed in the third trimester of pregnancy. Incidence of syphilis has been on the increase among young people in the recent years, and we anticipate that similar cases may increase in the future. Therefore, it is desirable to establish guidelines for management and mode of delivery of patients with syphilis in the second and third trimesters of pregnancy.

## AUTHOR CONTRIBUTIONS

**Ryosuke Tajiri:** Conceptualization; data curation; formal analysis; validation; visualization; writing – original draft. **Emi Kondo:** Supervision; writing – review and editing. **Yuma Saito:** Formal analysis; investigation. **Mao Sekimata:** Investigation; writing – review and editing. **Yasuyuki Kinjo:** Writing – review and editing. **Hiroshi Mori:** Supervision; writing – review and editing. **Kiyoshi Yoshino:** Supervision; writing – review and editing.

## FUNDING INFORMATION

No funding was received for conducting this study.

## CONFLICT OF INTEREST STATEMENT

The authors have no conflicts of interest directly relevant to the content of this article.

## ETHICS STATEMENT

This case report is not reviewed by the IRB as there are no identifying patient factors.

## CONSENT

Written informed consent was obtained from the patient to publish this report in accordance with the journal's patient consent policy.

## Data Availability

Data sharing is not applicable to this article as no new data were created or analyzed in this study.
